# CellSpatialGraph: Integrate hierarchical phenotyping and graph modeling to characterize spatial architecture in tumor microenvironment on digital pathology

**DOI:** 10.1016/j.simpa.2021.100156

**Published:** 2021-10-09

**Authors:** Pingjun Chen, Muhammad Aminu, Siba El Hussein, Joseph D. Khoury, Jia Wu

**Affiliations:** a Department of Imaging Physics, Division of Diagnostic Imaging, The University of Texas MD Anderson Cancer Center, Houston, TX, USA; b Department of Pathology, University of Rochester Medical Center, NY, USA; c Department of Hematopathology, Division of Pathology and Lab Medicine, The University of Texas MD Anderson Cancer Center, Houston, TX, USA

**Keywords:** Spatial analysis, Cell phenotyping, Graph modeling

## Abstract

We present CellSpatialGraph, an integrated clustering and graph-based framework, to investigate the cellular spatial structure. Due to the lack of a clear understanding of the cell subtypes in the tumor microenvironment, unsupervised learning is applied to uncover cell phenotypes. Then, we build local cell graphs, referred to as supercells, to model the cell-to-cell relationships at a local scale. After that, we apply clustering again to identify the subtypes of supercells. In the end, we build a global graph to summarize supercell-to-supercell interactions, from which we extract features to classify different disease subtypes.

**Table:** 

Code metadata	
Current code version	MICCAI21
Permanent link to code/repository used for this code version	https://github.com/SoftwareImpacts/SIMPAC-2021-118
Permanent link to Reproducible Capsule	https://codeocean.com/capsule/4501066/tree/v1
Legal Code License	BSD 3-Clause “New” or “Revised” License
Code versioning system used	git
Software code languages, tools, and services used	Matlab
Compilation requirements, operating environments & dependencies If available Link to developer documentation/manual	https://github.com/WuLabMDA/HierarchicalGraphModeling#readme
Support email for questions	jwu11@mdanderson.org

## 1. Introduction

The tumor is a complex ecosystem that emerges and evolves under selective pressure from its microenvironment, involving trophic, metabolic, immunological, and therapeutic factors. The relative influence of these biological factors orchestrates the abundance, localization, and functional orientation of cellular components within the tumor microenvironment (TME) with resultant phenotypic and geospatial variations, a phenomenon known as intratumoral heterogeneity [1]. With the advent of digital pathology, machine learning empowered computational pipelines have been proposed to profile intratumoral heterogeneity with H&E tissue sections to enhance cancer diagnosis and prognostication [2–4].

Most studies phenotype the textural patterns of tissue slides in a top-down manner with the deep convolutional neural networks (CNN) to extract versatile features tailored specifically for particular clinical scenarios [5–8]. Though these studies have achieved promising performance, they ignore the connections among individual cellular components and face challenges in biological interpretation. A few bottom-up studies focused on profiling cellular architectures from digital pathology slides have emerged using the graph theory approach and graph convolution network (GCN) approach [9–13]. The graph theory approach first constructs either local or global graph structures and then extracts hand-crafted features to test their clinical relevance. By contrast, the GCN approach aims to automatically learn representations from the global graph formed at the cellular level and abstract the features. However, a common limitation to these algorithms is their lack of ability to interpret the spatial patterns among different cellular levels.

To address these limitations, we propose a new computational framework that integrates graph modeling and unsupervised clustering algorithms to hierarchically decode cellular and clonal level pheno-types, explore their spatial patterns, and wrap up as CellSpatialGraph. In particular, we dissect the process into four key steps. First, we segment each cell and based on their features to identify intrinsic subtypes. Second, we focus on spatial interaction among neighboring cells via building local graphs factoring in their subtypes so that closely interacting cells are merged to form supercells. Third, we pool the supercells together to discover the cellular community at a population level. At last, we build global graphs incorporating community information to extract features for disease diagnosis purposes. We expect this framework can serve the research community to facilitate the in-depth understanding of intratumoral heterogeneity.

## 2. Description

### 2.1. Framework modules

This proposed framework in CellSpatialGraph mainly comprises four modules. In the “Cell Phenotyping via Unsupervised Learning” module, cells are segmented with a combination of multi-pass adaptive voting and local optimal threshold method [14,15]. Then the pheno-types of the cells are identified by their appearance features via the unsupervised clustering. In the “Supercell via Local Graph” module, we focus on spatial interaction among neighboring cells by building local graphs factoring in their subtypes so that closely interacting cells are merged to form supercells. Next, in the “Cell Community Identification by Clustering of Supercells” module, we pool the supercells together and apply spectral clustering to discover the cellular community at a population level. In the “Global Supercell Graph Construction and Feature Extraction”, we build global graphs incorporating community information to extract supercell interacting features for diagnosis purposes. CellSpatialGraph is written with Matlab and applicable across different operating systems, including Windows, macOS, and Linux.

### 2.2. Benchmark

We conduct the benchmark experiment on lymphoid neoplasms to test the proposed framework’s performance in diagnosing three hematological malignancy subtypes [16]. We compare with three cell-level graph-based algorithms, including the Global Cell Graph (GCG) [9], Local Cell Graph (LCG) [10], and FLocK [11]. The comparison results are shown in Table 1. The proposed framework shows superior performance on two evaluation metrics, including accuracy and area under the receiver operating characteristic curve (AUC), among the compared methods. The preliminary data suggests that our proposed hierarchical graph-based framework can better profile the multi-scale (both local and global) cellular interactions and intratumoral heterogeneity.

## 3. Impact

CellSpatialGraph is an open-source graph-based cell spatial analysis framework that provides a modularized pipeline to study the cellular spatial patterns to advance our understanding of intratumoral heterogeneity. This framework is among the first to integrate local and global graph approaches to interrogate cellular patterns within TME, and demonstrates superior performance in the diagnosis of lymphoid neoplasms [16]. Hereby, we hypothesize that the proposed design can overcome the limitations inherent in solely adopting either the global or local graph approaches, and conduct a more robust profiling intratumoral heterogeneity.

Besides, the clustering algorithms are employed to obtain the phenotypes at both cell and supercell (cell community) levels, given that their cellular components in TME are still under investigation. The unsupervised manner would shed light on uncovering new insight into biological subtypes of heterogeneous cells and clones.

## Figures and Tables

**Table 1:** Performance of the three compared algorithms and the proposed framework.

Method	Accuracy	AUC (CLL)	AUC (aCLL)	AUC (RT-DLBL)
GCG [9]	0.436 ± 0.037	0.421 ± 0.054	0.730 ± 0.027	0.770 ± 0.023
LCG [10]	0.471 ± 0.042	0.555 ± 0.049	0.669 ± 0.050	0.763 ± 0.032
FLocK [11]	0.601 ± 0.045	0.545 ± 0.054	**0.816** ± 0.025	0.847 ± 0.022
Proposed	**0.703** ± 0.030	**0.915** ± 0.009	0.724 ± 0.033	**0.866** ± 0.028
